# Correlation between Clinical Characteristics and Antibody Levels in COVID-19 Convalescent Plasma Donor Candidates

**DOI:** 10.3390/v15061357

**Published:** 2023-06-12

**Authors:** Günalp Uzun, Rebecca Müller, Karina Althaus, Matthias Becker, Patrick Marsall, Daniel Junker, Stefanie Nowak-Harnau, Nicole Schneiderhan-Marra, Harald Klüter, Hubert Schrezenmeier, Peter Bugert, Tamam Bakchoul

**Affiliations:** 1 Centre for Clinical Transfusion Medicine, University Hospital of Tuebingen, 72072 Tuebingen, Germany; guenalp.uzun@med.uni-tuebingen.de (G.U.); karina.althaus@med.uni-tuebingen.de (K.A.); stefanie.nowak-harnau@med.uni-tuebingen.de (S.N.-H.); 2 Institute for Clinical and Experimental Transfusion Medicine, Medical Faculty of Tuebingen, University Hospital of Tuebingen, 72072 Tuebingen, Germany; 3 Institute of Transfusion Medicine and Immunology, Medical Faculty Mannheim, Heidelberg University, German Red Cross Blood Service Baden-Württemberg-Hessen, 68167 Mannheim, Germany; r.mueller@blutspende.de (R.M.); harald.klueter@medma.uni-heidelberg.de (H.K.); peter.bugert@medma.uni-heidelberg.de (P.B.); 4 NMI Natural and Medical Sciences Institute at the University of Tuebingen, 72770 Reutlingen, Germany; matthias.becker@nmi.de (M.B.); patrick.marsall@nmi.de (P.M.); daniel.junker@nmi.de (D.J.); nicole.schneiderhan@nmi.de (N.S.-M.); 5 Institute for Clinical Transfusion Medicine and Immunogenetics Ulm, German Red Cross Blood Transfusion Service Baden-Württemberg-Hessen, 89081 Ulm, Germany; h.schrezenmeier@blutspende.de; 6 Institute for Transfusion Medicine and University Hospital Ulm, University of Ulm, 89081 Ulm, Germany

**Keywords:** COVID-19 convalescent plasma, neutralizing antibody, SARS-CoV-2

## Abstract

COVID-19 convalescent plasma (CCP) with high neutralizing antibodies has been suggested in preventing disease progression in COVID-19. In this study, we investigated the relationship between clinical donor characteristics and neutralizing anti-SARS-CoV-2 antibodies in CCP donors. COVID-19 convalescent plasma donors were included into the study. Clinical parameters were recorded and anti-SARS-CoV-2 antibody levels (Spike Trimer, Receptor Binding Domain (RBD), S1, S2 and nucleocapsid protein) as well as ACE2 binding inhibition were measured. An ACE2 binding inhibition < 20% was defined as an inadequate neutralization capacity. Univariate and multivariable logistic regression analysis was used to detect the predictors of inadequate neutralization capacity. Ninety-one CCP donors (56 female; 61%) were analyzed. A robust correlation between all SARS-CoV-2 IgG antibodies and ACE2 binding inhibition, as well as a positive correlation between donor age, body mass index, and a negative correlation between time since symptom onset and antibody levels were found. We identified time since symptom onset, normal body mass index (BMI), and the absence of high fever as independent predictors of inadequate neutralization capacity. Gender, duration of symptoms, and number of symptoms were not associated with SARS-CoV-2 IgG antibody levels or neutralization. Neutralizing capacity was correlated with SARS-CoV-2 IgG antibodies and associated with time since symptom onset, BMI, and fever. These clinical parameters can be easily incorporated into the preselection of CCP donors.

## 1. Introduction

The COVID-19 pandemic has affected every aspect of life and is still going on three years after it began. The world has been caught off guard by the COVID-19 pandemic [1]. Initially, there was little understanding of SARS-CoV-2 and its associated respiratory infection, and no treatment was available. Passive immunization using convalescent plasma seems to be an attractive treatment option. The concept of convalescent plasma therapy is to transfer antibodies from individuals who have successfully recovered from a particular disease to individuals currently fighting the same infection [2]. Plasma collected from recovered individuals contains these antibodies, which can help neutralize the pathogen and boost the recipient’s immune response [3]. In addition to being readily available as soon as people recover from their illness, another advantage of convalescent plasma is that the existing infrastructure and protocols of blood services can be easily used to collect and distribute convalescent plasma [4,5].

Based mainly on a theoretical background and some small case series with SARS infection [6,7], the US Food and Drug Administration (FDA) has granted an emergency use authorization for convalescent plasma therapy among other experimental treatments in patients with COVID-19 [8]. Similarly, the European Commission has funded 24 projects for a total of €36 million to develop or expand programs to collect plasma from COVID-19-recovered donors [9].

The use of COVID-19 convalescent plasma (CCP) in patients with COVID-19 was then evaluated in a series of randomized clinical trials [10]. Although some showed no benefit from CCP in COVID-19, other studies showed the beneficial effects of plasma products with high neutralizing antibody titers [10,11,12,13,14,15]. Furthermore, a recent guideline from the Association for the Advancement of Blood and Biotherapies (AABB) suggests CCP in outpatients with high risk for disease progression and in hospitalized patients lacking SARS-CoV-2 antibodies at admission [16]. The effectiveness of the CCP therapy depends on the number of neutralizing antibodies in the product [11,12]. Accordingly, the revised FDA approval also specifies the use of high neutralizing antibody titers only [17]. However, data from the European Blood Bank Central Registry indicate that only one-third of blood donations meet the eligibility criteria for transfusion [18]. This means that a significant portion of CCP products cannot be utilized for treating COVID-19 patients. Consequently, the collection and long-term storage of these products result in unnecessary additional costs.

The lack of a standardized donor selection criteria for individuals with high neutralizing antibody titers further complicates the situation [18]. Clinical selection criteria for donors with high neutralizing antibody titers have yet to be defined [2]. Defining these criteria would enable the identification of suitable donors and improve the efficiency of CCP collection, ensuring that the available resources are focused on obtaining CCP products with the highest therapeutic potential.

This study investigates the relationship between clinical and temporal parameters and anti-SARS-CoV-2 antibodies in unvaccinated CCP donors.

## 2. Materials and Methods

### 2.1. Study Cohort

Patients who had recovered from COVID-19 were invited through the website of the German Red Cross to donate convalescent plasma. Additionally, CCP donor candidates were asked to participate in the CORE trial [19,20]. To ensure the suitability of potential plasma donors, their availability for donation was evaluated based on the prevailing guidelines of the German regulatory authorities. Donors were required to have been free from any symptoms related to SARS-CoV-2 for a minimum of two weeks. The infection was documented either by a positive SARS-CoV-2 polymerase chain reaction (PCR) test (by nasal or pharyngeal swab), antibody enzyme immunoassay or a rapid antigen test. Over the course of six months, from January 2021 to June 2021, a total of 115 convalescent donors were recruited from two blood donation centers: the Centre for Clinical Transfusion Medicine in Tübingen, Germany, and the Institute of Transfusion Medicine and Immunology in Mannheim, Germany. All of these donors underwent a SARS-CoV-2 antigen test prior to their blood donation, which confirmed that they were not carrying the virus at the time of donation. To gather essential information about the donors, a comprehensive questionnaire was administered, covering baseline characteristics, medical history, and the presence of any COVID-19 symptoms. Additionally, blood samples were collected from the donors before their plasma donation, and these samples were appropriately frozen for subsequent analysis.

### 2.2. Measurement of SARS-CoV-2 Antibodies

COVID-19 antibodies were measured with a multiplex serological assay (MULTICOV AB, NMI, Reutlingen, Germany) with the FLEXMAP 3D^®^ system (Luminex Corporation, Austin, TX, USA), as published previously [21]. In brief, 25 μL of the 1:200 diluted samples were incubated with a 25 µL bead mix for 2 h at 21 °C in a 96-well plate (#3642, Corning, Somerville, MA, USA) on a thermomixer (Eppendorf, Hamburg, Germany). Beads were washed three times with 100 µL of wash buffer (1× PBS, 0.05% (*v*/*v*) Tween20) using a microplate washer with a magnetic plate separator (Biotek 405TS, Biotek Instruments GmbH, Friedrichshall, Germany) to remove unbound antibodies. R-phycoerythrin (RPE)-labeled goat-anti-human IgG (#109-116-098, Dianova, 3 µg/mL, Hamburg, Germany) antibodies were added and the plate was incubated for 45 min at 21 °C on a thermomixer and washed three times afterwards, as described before. Quality control samples were processed on each plate in duplicate. Plates were measured using a Luminex FLEXMAP 3D instrument and the Luminex xPONENT Software 4.3 (with the following settings: sample size, 80 µL, 50 events; gate, 7500–15,000; reporter gain, Standard PMT). Antibody levels were normalized by dividing the median fluorescence intensity (MFI) values of each sample by the mean MFI value of the quality control samples for each antigen separately. A normalized MFI value > 1 for both the trimeric Spike and RBD indicates positivity in SARS-CoV-2 antibody measurements.

### 2.3. Measurement of ACE2 Binding Inhibition by RBD Antibodies

ACE2 binding inhibition was measured using a bead-based multiplex RBDCoV-ACE2 assay, as previously described [22]. Biotinylated human ACE2 (Sino Biological, # 10108-H08H-B, Beijing, China) was added to the assay buffer for a final concentration of 342.9 ng/mL to prepare ACE2 buffer. Serum samples were thawed and diluted 1:25 in assay buffer before being further diluted 1:8 in ACE2 buffer, resulting in a final concentration of 300 ng/mL ACE2 in all samples diluted 1:200. A bead mix was prepared using MagPlex beads (Luminex, 40 beads/µL per bead population) coupled to wild-type SARS-CoV-2 RBD protein. Diluted serum (25 µL) and bead mixture (25 µL) were added to each well of a 96-well plate (Corning, #3642). A total of 300 ng/mL ACE2 was added to one well instead of serum and measured in duplicate on each plate as a normalization control. In addition, two quality control samples were assayed in duplicate on each plate. For blank measurement, 25 µL of assay buffer was added to two wells of each plate instead of the diluted sample. The assay plate was then placed on a thermomixer at 750 rpm and incubated at 21 °C for 2 h. The beads were washed three times with 100 µL of wash buffer (1× PBS, 0.05% (*v*/*v*) Tween20) using a microplate washer (Biotek 405TS, Biotek Instruments GmbH). RPE-Streptavidin (30 µL at 2 µg/mL) was added to each well to detect biotinylated bound ACE2. The plate was then incubated for 45 min at 21 °C on a thermal mixer. The beads were washed three times with 100 µL wash buffer. Plates were measured using a Luminex FLEXMAP 3D instrument and Luminex xPONENT software 4.3 (the settings were as follows: sample size, 80 µL, 50 events; gate, 7500–15,000; reporter gain, Standard PMT). The MFI values of each sample were divided by the mean value of the ACE2 normalization control. The normalized values were converted to percentages and subtracted from 100 to obtain the percentage of ACE2 binding inhibition. Negative values were set to zero. The determination of the cut-off for the ACE2 binding inhibition assay was based on a previous study conducted by Junker et al. [22]. In their research, Junker et al. compared RBDCoV-ACE2 with the virus neutralization test and found a strong correlation between the two tests. They observed that samples with an ACE2 binding inhibition below 20% had significantly lower VNT50 values, indicating reduced neutralization activity. Furthermore, a commercially available ACE2 inhibition test (SARS-CoV-2 NeutraLISA, Euroimmun AG, Lübeck, Germany) also utilized a 20% cut-off to distinguish between positive and negative samples. A comparison between the RBDCoV-ACE2 assay and the NeutraLISA assay demonstrated a high correlation between the two. In light of these findings, they recommended the <20% ACE2 inhibition threshold as the criterion for considering a sample as negative [22].

### 2.4. Ethics Statement

The study was conducted in accordance with the Declaration of Helsinki. Written informed consent was obtained from all blood donors prior to any study-related procedure. The study protocol was approved by the Ethics Committee at the Medical Faculty of the Eberhard Karls University and at the University Hospital Tübingen (897/2020BO2) and the Ethical Committee of the Medical Faculty Mannheim (2020-643N).

### 2.5. Statistics

Statistical analyses were performed with GraphPad Prism 9.4.1 (La Jolla, CA, USA). The normality of the data was assessed using the D’Agostino and Pearson omnibus normality test. If the data were normally distributed, a *t*-test was used to analyze the results. If the data did not follow a normal distribution, the Mann–Whitney test was used. Spearman’s correlation was used to evaluate the association between antibody levels and continuous clinical parameters. Univariate and multiple binary logistic regression analysis was performed using SPSS Version 29 (IBM Inc., Armonk, NY, USA) to identify independent predictors of factors associated with inadequate neutralization. Odds ratios (OR) with 95% confidence intervals (CI) are reported. A *p*-value less than 0.05 was considered statistically significant.

## 3. Results

### 3.1. Study Cohort

In this study, a total of 115 CCP donor candidates were enrolled. However, four donors had to be excluded later due to negative results on the SARS-CoV-2 antibody test, and three donors were excluded because of missing clinical data. Additionally, 17 donors who had received a vaccine prior to plasma donation were also excluded from the analysis. Therefore, the remaining 91 CCP donors (56 female, 35 male), with a median age of 40.0 years (range, 20–60), were included in the analysis. Of these donors, 77 (85%) were diagnosed with COVID-19 using a PCR test, 10 (11%) using an antibody test, and 4 (4%) using a rapid antigen test. The median duration from the start of COVID-19 infection was 113 days (range, 25–442) and the median duration of symptomatic disease was eight days (range, 0–75). Table 1 presents the characteristics of the patients.

### 3.2. Correlation between Donor Characteristics and SARS-CoV-2 Antibody Levels

**Figure 1:**
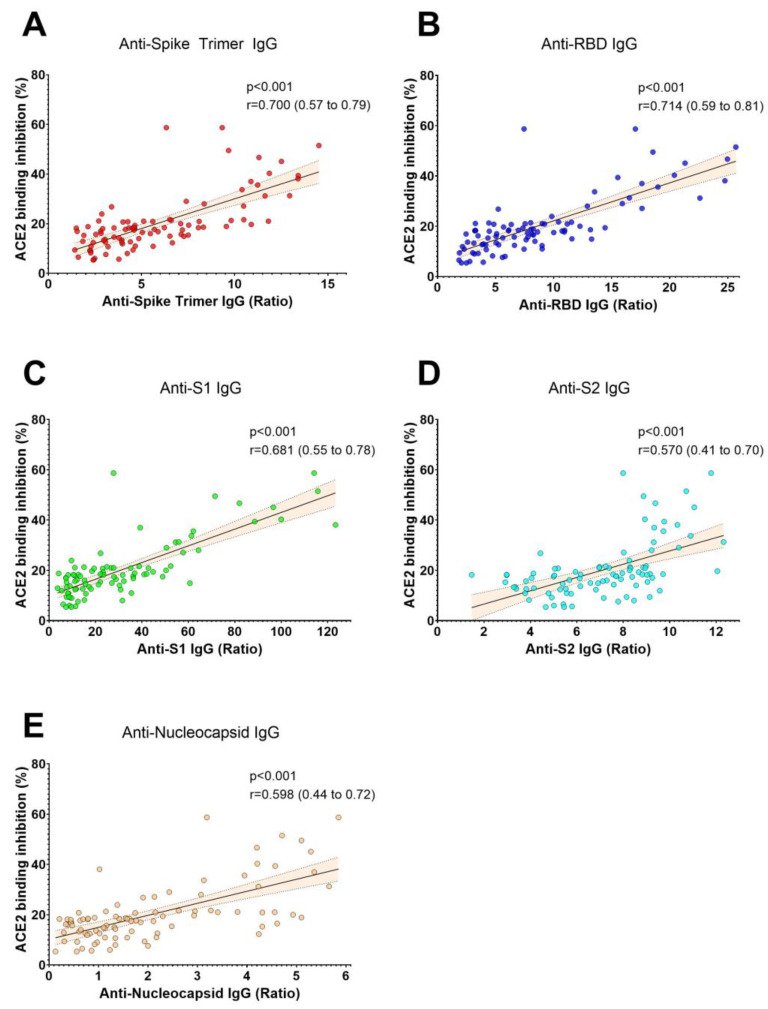
The correlation between SARS-CoV-2 antibody levels and ACE2 binding inhibition. ACE2 binding inhibition was strongly correlated with the levels of anti-SARS-CoV-2 IgG antibodies specific for Spike Trimer (**A**), Receptor Binding Domain (RBD) (**B**), S1 (**C**), and moderately correlated with the levels of anti-SARS-CoV-2 IgG antibodies specific for S2 (**D**) and nucleocapsid protein (**E**) in non-vaccinated COVID-19 convalescent plasma donors (*n* = 91). Spearman’s rank correlation coefficients (r) and *p* values are presented in each panel. The dashed area represents the 95% confidence interval.

We measured SARS-CoV-2 IgG antibodies against five different antigens and ACE2 binding inhibition in samples from CCP donor candidates (Table 1). Our findings indicate a robust correlation between the levels of different SARS-CoV-2 antibodies and ACE2 binding inhibition (Figure 1, Table 2). Additionally, we found that the length of time from the start of infection until blood donation was negatively correlated with all SARS-CoV-2 antibody levels, whereas the total number of symptoms was only correlated with S2 IgG antibody levels (Table 2). Notably, the duration of symptomatic disease was not correlated with antibody levels or ACE2 binding inhibition (Table 2). Furthermore, the donor age was positively correlated with all antibody levels (Table 2). Finally, we found that BMI showed a positive correlation with antibody levels (Table 2).

### 3.3. Predictors of Inadequate SARS-CoV-2 Antibody Neutralization

**Figure 2:**
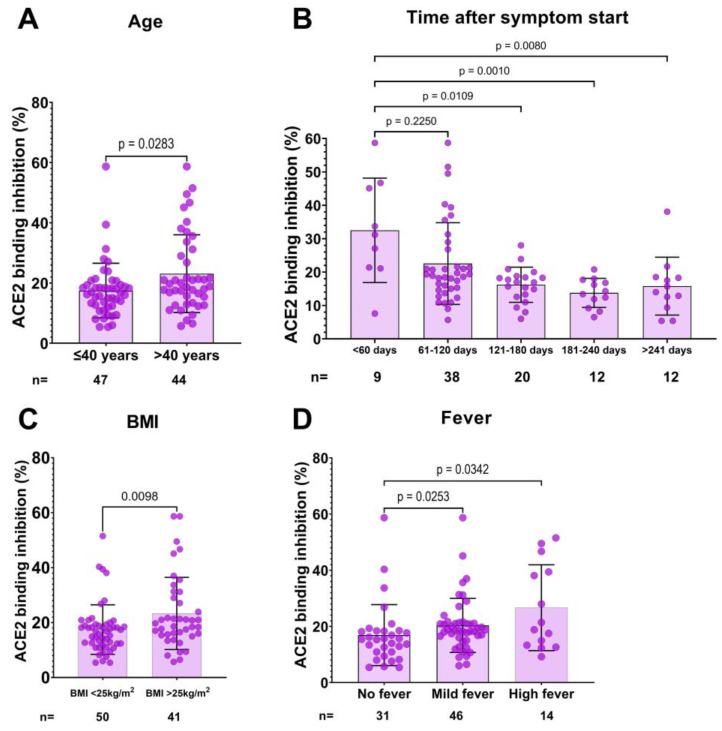
Association between ACE2 binding inhibition and donor characteristics. Donors over 40 years of age had a significantly higher neutralization capacity compared to younger donors (**A**). Donors who donated plasma within 60 days of infection had a higher neutralization capacity compared to those who donated later than 120 days after infection (**B**). A body mass index (BMI) > 25 was associated with a higher neutralization capacity (**C**). Donors without a fever had a significantly lower neutralization capacity compared to donors with a fever (**D**). Statistical analysis was performed using either the Mann–Whitney U test or the KruskalWallis Test. *p* values are indicated in each panel.

Logistic regression analysis was used to analyze the relationship between donor characteristics and inadequate neutralization capacity (<20%) in CCP donor candidates (Table 3). In univariate logistic regression, we found that donor age (<40 years), BMI (<25 kg/m^2^), time after symptoms began (>120 days), and not having fever were associated with a higher chance of having inadequate neutralization capacity (Table 3, Figure 2). On the other hand, multiple logistic regression analyses revealed that the time after symptoms began (*p* = 0.003, OR (95% CI) = 6.09 (1.88–19.68)), normal BMI (<25 kg/m^2^) (*p* = 0.029, OR (95% CI) = 3.33 (1.13–9.80)) and not having high fever (*p* = 0.020, OR (95% CI) = 7.22 (1.36–38.26)) are independent predictors of inadequate neutralization capacity.

## 4. Discussion

In the current study, we measured antibody levels in unvaccinated CCP donor candidates. We found high individual variability in terms of SARS-CoV-2 antibody levels. As reported in previous studies, a considerable proportion of donor candidates did not demonstrate adequate neutralizing antibodies [23,24]. Therefore, the implementation of a robust preselection algorithm is crucial to exclude individuals who lack an adequate antibody response. In the current study, time after symptom onset, high fever and BMI were independently associated with the neutralization capacity of SARS-CoV-2 antibodies.

Upon exposure to viral components, B cells proliferate and produce antibodies that recognize viral antigens and neutralize the virus. In the case of SARS-CoV-2, IgG antibodies can be detected within two weeks after infection, and their levels rise to a peak between three and seven weeks after the onset of symptoms [25]. After the peak, the levels of IgG antibodies remain stable for some time before gradually decreasing [25,26]. Boonyaratanakornkit et al. calculated a half-life of 66 days for neutralizing antibodies in CCP donors [27]. Similarly, we found that, compared to donations within 60 days post-infection, neutralization capacity was significantly lower in donations 120 days or longer post infection. Therefore, it may be beneficial to prioritize plasma donations from those who were infected more recently, as they may be more likely to have higher neutralizing antibody levels.

Our analysis has revealed, a correlation, albeit a weak one, between donor age and both SARS-CoV-2 antibody levels and neutralizing capacity. This is consistent with previous research indicating that older individuals tend to produce higher levels of antibodies following COVID-19 infection than younger individuals [27,28,29]. However, not all studies have found a correlation between age and antibody levels [23,30]. It is worth noting that our multivariable analysis also showed that donor age was not associated with neutralizing capacity. Regarding the influence of donor sex, our findings are in line with prior studies that have shown no significant association between gender and SARS-CoV-2 antibody levels [23,24,31]. 

Several studies have examined the relationship between infection severity and antibody levels in CCP donors. Generally, hospitalized patients exhibit higher antibody levels than convalescent outpatients [21,24,27,29,32], and other studies have demonstrated a positive correlation either between individual symptoms or between the number of symptoms and neutralizing antibody titers [23,31]. Bosnjak et al. found a weak correlation between the duration of symptomatic disease, and SARS-CoV-2 S1 antibodies and neutralizing antibody titers [30]. It is important to note that antibody response is delayed in patients with severe COVID-19 [33]. However, Parker et al. found that although antibody levels in patients with severe disease began to rise one to two weeks later than in patients with moderate disease, they later reached similar levels [34]. Recently, Körper et al. reported a trend toward a better outcome in patients receiving CCP from donors with more than three symptoms compared to controls, but the difference was not significant (*p* = 0.061) [12]. In the current study, we did not find a correlation between neutralizing antibody levels and the number of reported symptoms, as well as the duration of the infection among CCP donor candidates. Instead, we found a positive association between fever and higher neutralizing antibody levels. Others also found that fever was associated with higher antibody levels and neutralizing antibody titers [24,31,35]. Fever is a common symptom of infection, and triggers a series of responses both in innate and adaptive immunity that help the body fight off the invading pathogen [36]. Thus, individuals with fever may produce higher levels of antibodies in response to an infection. We propose that fever is a criterion that is easy to apply and could be easily incorporated into algorithms for the selection of donors.

In our study, we found that individuals with a BMI below 25 are more likely to have inadequate levels of neutralizing antibodies. This agrees with the findings of Zhai et al. who reported a positive correlation between BMI and serum antibody levels among COVID-19 convalescent outpatients [37]. Additionally, a large-scale study of over 12,000 convalescent individuals found a strong association between obesity and neutralizing antibody response following COVID-19 infection [38]. This may be due to the fact that patients with obesity tend to experience more severe infections, which leads to higher levels of neutralizing antibodies [39]. Conversely, other research has suggested that individuals with a high BMI may have a weaker immune response to COVID-19 infections [39], likely due to changes in the immune system and altered antibody production caused by the chronic inflammation associated with obesity [40]. To gain a better understanding of how BMI impacts antibody production, particularly in regard to neutralizing antibodies after infection, additional studies are necessary.

This study has a number of limitations. First, we used a bead-based ACE2-RBD binding inhibition assay to measure neutralizing antibodies in CCP donors. The virus neutralization assay is the gold standard for measuring neutralizing antibody titers, but this test is not widely available due to its high technical requirements. The recently developed bead-based multiplex assay for ACE2-RBD binding inhibition is designed to detect the relative reduction in ACE2-RBD binding caused by neutralizing antibodies present in the sample. Furthermore, a high correlation between the virus neutralization assay and the ACE2-RBD binding inhibition assay has been demonstrated. Second, our study cohort did not include hospitalized COVID-19 convalescents. It has been reported that neutralizing antibody levels are higher in hospitalized patients [23,24,32]. The vast majority of convalescents, however, are not hospitalized, and previous studies have shown that hospitalized COVID-19 convalescents represent a small minority of CCP donor candidates [27]. Therefore, identifying predictors of higher neutralizing antibody levels in this cohort is of clinical importance. Logically, hospitalized COVID-19 convalescents may have comorbidities that may exclude them from plasma donation due to the strict regularities of blood donation. Furthermore, the neutralization of antibodies in CCP might diminish against new emerging virus variants and, therefore, the association between clinical parameters and neutralization against new variants might be different. Further studies investigating the validity of these predictors by other virus variants are definitely warranted. Finally, we excluded vaccinated donor candidates because this allowed a more focused analysis of the association between clinical and demographic characteristics and neutralizing antibody levels in convalescent plasma donors without the potential confounding effects of vaccination. Neutralizing antibody levels in plasma donors may be affected by the timing of vaccination. Antibody levels may be highest shortly after vaccination and then gradually decline over time, which could potentially bias the results if vaccinated individuals were included in the study.

## 5. Conclusions

Looking forward, the COVID-19 pandemic has taught us valuable lessons that we can use to prepare for future pandemics. In the early stages of a new outbreak, convalescent plasma may be the most readily available treatment option [10]. Furthermore, passive immunization with convalescent plasma will emerge as an attractive treatment option not only because of its ready availability once convalescents become available, its potential efficacy, and its ease of collection and distribution using the existing infrastructure of blood donation services. In this regard, our results may help to select donors with potentially higher neutralizing antibodies where antibody or neutralization testing is not available. This approach can reduce the unnecessary collection and storage of plasma products with low neutralizing antibody titers, and ultimately save costs.

## Figures and Tables

**Table 1:** Characteristics of COVID-19 convalescent plasma donor candidates (*n* = 91). RBD = receptor binding domain.

Patient Characteristic	Median (Range) or *n* (%)
Median age in years (range)	40 (20–60)
Female/Male (%)	56 (61.5%)/35 (38.5%)
Median body mass index in kg/cm^2^ (range)	24.7 (18.3–46.8)
Blood group, *n* (%)	
O	35 (38.5%)
A	44 (48.4%)
B	8 (8.8%)
AB	4 (4.4%)
Median duration of symptoms in days (range)	8 (0–75)
Median time after symptom onset in days (range)	113 (25–442)
Median SARS-CoV-2 Spike Trimer IgG ratio (range)	4.6 (1.4–14.5)
Median SARS-CoV-2 RBD IgG ratio (range)	7.5 (1.85–25.6)
Median SARS-CoV-2 S1 IgG ratio (range)	22.9 (3.7–123.3)
Median SARS-CoV-2 S2 IgG ratio (range)	7.4 (1.5–12.3)
Median SARS-CoV-2 Nucleocapsid IgG ratio (range)	1.6 (0.2–5.9)
Median ACE2 binding inhibition in % (range)	18 (5.4–58.7)
Clinical symptoms, *n* (%)	
Mild fever (37.5–38.5 °C)	46 (50.5%)
High fever (>39.5 °C)	14 (15.4%)
Anosmia/dysgeusia	62 (68.1%)
Headache	68 (74.4%)
Sore throat	48 (52.7%)
Rhinitis	49 (53.8%)
Cough	53 (58.9%)
Dyspnea	39 (43.3%)
Pneumonia	9 (9.9%)
Fatigue	77 (84.6%)
Muscle/joint pain	64 (70.3%)
Nausea/diarrhea	17 (18.7%)
Eye	7 (7.7%)
Skin symptoms	4 (4.4%)
Ear	10 (11.1%)

**Table 2:** Correlation between SARS-CoV-2 IgG antibody levels, ACE2 binding inhibition and clinical parameters.

Parameter		Anti-Spike Trimer IgG	Anti-RBD IgG	Anti-S1 IgG	Anti-S2 IgG	Anti-Nucleocapsid IgG	ACE2 Binding Inhibition
Age	Spearmen r	0.279 *	0.265 *	0.230 *	0.314 *	0.417 *	0.273 *
	*p*	0.01	0.01	0.03	<0.001	<0.001	0.01
Body mass index	Spearmen r	0.379 *	0.293 *	0.341 *	0.438 *	0.363 *	0.296 *
	*p*	<0.001	<0.001	<0.001	<0.001	<0.001	<0.001
Duration of symptoms	Spearmen r	0.13	0.13	0.17	0.13	0.11	0.12
	*p*	0.21	0.21	0.11	0.22	0.32	0.25
Time after symptom begin	Spearmen r	−0.397 *	−0.386 *	−0.336 *	−0.276 *	−0.582 *	−0.417 *
	*p*	<0.001	<0.001	<0.001	0.01	<0.001	<0.001
Total number of symptoms	Spearmen r	0.20	0.18	0.14	0.252 *	0.09	0.13
	*p*	0.06	0.09	0.17	0.02	0.38	0.20
ACE2 binding inhibition	Spearmen r	0.700 *	0.714 *	0.681 *	0.570 *	0.598 *	-
	*p*	<0.001	<0.001	<0.001	<0.001	<0.001	-

(*) denotes a statistically significant correlation.

**Table 3:** Logistic regression model of predictors of inadequate naturalization capacity.

	ACE2 Binding Inhibition		Univariate Analysis				Multiple Regression Analysis			
				95% CI				95% CI		
	<20% (*n* = 61)	≥20% (*n* = 30)	Odds Ratio	Lower	Upper	*p*	Odds Ratio	Lower	Upper	*p*
Age < 40 years	24 (39%)	20 (67%)	3.08	1.23	7.71	0.016 *	2.15	0.74	6.21	0.159
Female vs. Male	40 (66%)	16 (53%)	1.67	0.68	4.06	0.261				
Body mass index (BMI) < 25 kg/m^2^	40 (66%)	10 (33%)	3.81	1.51	9.61	0.005 *	3.33	1.13	9.80	0.029 *
Blood group (A vs. O) ^†^	26 (48%)	18 (72%)	0.36	0.13	1.01	0.051				
Duration of symptoms > 7 days	31 (51%)	19 (63%)	1.67	0.68	4.10	0.261				
Time after symptom begin > 120 days	38 (62%)	6 (20%)	6.61	2.35	18.58	<0.001 *	6.09	1.88	19.68	0.003 *
Total number of symptoms ≤ 6	34 (58%)	12 (40%)	1.89	0.78	4.59	0.160				
no fever vs. high fever	7 (12%)	7 (23%)	5.20	1.26	21.49	0.023 *	7.22	1.36	38.26	0.020 *
no fever vs. mild fever	28 (46%)	18 (60%)	3.34	1.08	10.30	0.036 *	2.03	0.57	7.19	0.273
Anosmia/dysgeusia	41 (67%)	21 (70%)	0.88	0.34	2.26	0.789				
Headache	45 (74%)	23 (77%)	0.86	0.31	2.38	0.765				
Sore throat	33 (54%)	15 (50%)	1.18	0.49	2.83	0.713				
Rhinitis	34 (56%)	15 (50%)	1.26	0.52	3.02	0.606				
Cough	37 (61%)	17 (57%)	1.18	0.49	2.86	0.716				
Dyspnea	25 (41%)	14 (47%)	0.79	0.33	1.91	0.607				
Pneumonia	5 (8%)	4 (13%)	0.58	0.14	2.34	0.444				
Fatigue	50 (82%)	27 (90%)	0.51	0.13	1.97	0.325				
Muscle/joint pain	42 (70%)	22 (73%)	0.80	0.30	2.13	0.660				
Nausea/diarrhea	12 (20%)	5 (17%)	1.22	0.39	3.86	0.730				
Ocular symptoms	4 (7%)	3 (10%)	0.63	0.13	3.02	0.565				
Skin symptoms	2 (3%)	2 (7%)	0.48	0.06	3.55	0.468				
Ear symptoms	5 (8%)	5 (17%)	0.45	0.12	1.68	0.233				

(*) denotes a statistically significant analysis, (^†^) Only donors with blood group A or O included in the analysis (*n* = 79).

## Data Availability

Data may be requested for academic collaboration from the corresponding author.
